# Glutamate carboxypeptidase II activation in astrocytes mediates glymphatic impairment and cognitive vulnerability in the aging brain following surgery

**DOI:** 10.1002/alz.71666

**Published:** 2026-07-09

**Authors:** Yuto Hasegawa, Hiroki Kawai, Robyn Wiseman, Gianluca Ursini, Hannah Alton, Feiyi Xiong, Sohan Gummadi, Naomi Mao, Kyle Mailhot, Yannan Li, Xiaolei Zhu, Tomoyo Sawada, Barbara Slusher, Atsushi Kamiya

**Affiliations:** ^1^ Department of Psychiatry and Behavioral Sciences Johns Hopkins University School of Medicine Baltimore Maryland USA; ^2^ Johns Hopkins Drug Discovery Johns Hopkins University School of Medicine Baltimore Maryland USA; ^3^ Department of Pharmacology and Molecular Sciences Johns Hopkins University School of Medicine Baltimore Maryland USA; ^4^ Lieber Institute for Brain Development Johns Hopkins Medical Campus Baltimore Maryland USA; ^5^ Department of Psychiatry, Friedman Brain Institute Icahn School of Medicine at Mount Sinai New York New York USA; ^6^ Department of Neurology Johns Hopkins University School of Medicine Baltimore Maryland USA; ^7^ Department of Neuroscience Johns Hopkins University School of Medicine Baltimore Maryland USA

**Keywords:** aging, astrocytes, glutamate carboxypeptidase II, glymphatic system, perioperative neurocognitive disorder, sex differences

## Abstract

**INTRODUCTION:**

Perioperative neurocognitive disorder is a common and debilitating complication in the elderly, yet its cellular and molecular mechanisms in the aging brain remain poorly understood.

**METHODS:**

Using aged mice, we examined the impact of abdominal surgery on cognition, glymphatic activity, and astrocyte function. Sex‐dependent mechanisms were investigated by integrating single‐cell RNA sequencing with astrocyte‐specific genetic and pharmacological manipulation.

**RESULTS:**

Abdominal surgery induced male‐specific deficits in recognition and spatial memory, reduced hippocampal glymphatic influx, and glutamate accumulation in aged mice. These changes were associated with male‐specific upregulation of glutamate signaling in a distinct astrocyte subpopulation, enhanced astrocytic glutamate carboxypeptidase II (GCPII) activity, and loss of aquaporin‐4 (AQP4) polarization. Astrocyte‐specific GCPII knockdown rescued cognitive deficits and hippocampal glymphatic influx, consistent with pharmacological GCPII inhibition ameliorating glutamate levels, AQP4 polarization, and cognitive performance.

**DISCUSSION:**

These findings identify astrocytic GCPII‐mediated glutamate dysregulation as a mechanism contributing to sex‐specific postoperative cognitive vulnerability in aging.

## Highlights

- Abdominal surgery induces male‐specific cognitive and glymphatic deficits in aged mice.
- Surgery disrupts perivascular astrocytes and aquaporin‐4 (AQP4) polarization in the aged hippocampus.
- A distinct astrocyte subpopulation exhibits male‐specific glutamate signaling changes.
- Astrocytic glutamate carboxypeptidase II (GCPII) mediates postoperative deficits in glymphatic influx and cognitive function.
- GCPII inhibition reduces excessive glutamate and ameliorates cognitive deficits.

## 1 BACKGROUND

Perioperative neurocognitive disorder (PND) is a common neuropsychiatric complication in older adults following surgery characterized by impairments across multiple cognitive domains, including learning and memory.^1^, ^2^, ^3^, ^4^, ^5^, ^6^ PND is associated with an increased risk of dementia.^3^, ^7^, ^8^ PND hinders postoperative recovery, increases mortality, and imposes significant socioeconomic burdens.^9^, ^10^, ^11^ Approximately 20% of older patients develop cognitive impairment within 1 week after major surgery,^12^ highlighting PND as a clinically relevant manifestation of cognitive vulnerability in the aging brain. Despite its substantial public health impact, the heterogeneous etiology of PND, which arises from a complex interplay of perioperative factors and individual vulnerabilities, continues to challenge our understanding of its underlying pathophysiology.^2^, ^3^, ^13^ PND has been reported in both men and women in clinical cohorts.^12^, ^14^, ^15^ Although sex is increasingly recognized as an important modifier of brain aging and cognitive function,^16^, ^17^ it remains unclear whether pathological processes implicated in PND are similarly engaged across sexes or exhibit sex‐dependent features.

Multiple preclinical models revealed that surgical interventions, such as abdominal surgery and tibial fracture, trigger systemic inflammation that induces neuroinflammation.^18^, ^19^, ^20^, ^21^, ^22^ These processes are mediated by circulating pro‐inflammatory cytokines, chemokines, and monocytes, which interact with brain resident immune cells, leading to neuronal dysfunction and cognitive deficits.^20^, ^23^, ^24^, ^25^, ^26^, ^27^, ^28^, ^29^ Astrocytes, critical regulators of brain homeostasis, respond to inflammatory conditions by releasing gliotransmitters such as glutamate.^30^ Under physiological conditions, astrocytic glutamate transporters, including glutamate transporter 1 (GLT‐1) and glutamate–aspartate transporter (GLAST), maintain extracellular glutamate homeostasis, preventing excitotoxicity.^31^ However, excessive glutamate exposure induces reactive astrogliosis characterized by cellular hypertrophy, glial fibrillary acidic protein (GFAP) upregulation, and dysregulated calcium signaling.^32^ These changes impair neuronal function, contributing to cognitive deficits.^24^, ^33^, ^34^, ^35^ Emerging evidence further implicates astrocytes in glymphatic system, a waste clearance pathway that facilitates the removal of metabolic byproducts from the brain.^36^, ^37^, ^38^ Aquaporin‐4 (AQP4) channels, which are highly polarized on the perivascular endfeet of astrocytes, are essential for facilitating cerebrospinal fluid (CSF) flow and interstitial fluid exchange, thereby modulating glymphatic activity.^39^, ^40^, ^41^ Interestingly, glutamate has been shown to alter AQP4 expression, potentially disrupting glymphatic function.^42^ Recent studies further indicate that postoperative reductions in glymphatic activity correlate with cognitive decline,^43^, ^44^, ^45^ suggesting that impaired glymphatic system may contribute to postoperative cognitive vulnerability in the aging brain. Nevertheless, the molecular and cellular mechanisms of how surgical intervention alters astrocyte function and disrupts the glymphatic system, leading to cognitive impairments remain poorly understood.

In this study, we investigate whether astrocytic dysfunction underlies postoperative cognitive deficits in a sex‐dependent manner using an aged mouse model of abdominal surgery. We also examine how surgical stress alters astrocyte‐mediated regulation of glutamate homeostasis and glymphatic function, with particular focus on the role of glutamate carboxypeptidase II (GCPII), a key regulator of astrocytic glutamate production,^46^, ^47^ and determine whether genetic or pharmacological modulation of GCPII can mitigate surgery‐induced phenotypes. By integrating behavioral analyses with molecular, cellular, and single‐cell transcriptomic approaches in both male and female mice, this study aims to define sex‐specific astrocyte‐mediated mechanisms driving postoperative cognitive vulnerability and to identify potential therapeutic targets for preventing PND in aging populations.

**RESEARCH IN CONTEXT**

1. **Systematic review**: Perioperative neurocognitive disorder (PND) is a common complication in older adults and is associated with increased dementia risk. Prior studies implicate neuroinflammation, microglial activation, blood–brain barrier disruption, and impaired glymphatic clearance in PND. However, astrocyte‐specific molecular mechanisms linking surgical stress to glymphatic dysfunction and cognitive vulnerability in the aging brain remain poorly investigated.
2. **Interpretation**: This study identifies glutamate carboxypeptidase II (GCPII) activation as an astrocyte‐mediated mechanism underlying male‐specific postoperative glutamate dysregulation, impaired glymphatic influx, and cognitive deficits in aged mice. Male‐specific upregulation of glutamate signaling within a distinct astrocyte subpopulation further position astrocytic glutamate dysregulation as a sex‐specific contributor to postoperative cognitive vulnerability.
3. **Future directions**: Future studies should define upstream regulators of sex‐specific astrocyte dysfunction and develop GCPII‐targeted strategies to preserve glymphatic function and cognitive resilience during aging and surgical stress.

## 2 METHODS

### 2.1 Mice

C57BL/6J aged mice (18–20 months old) were obtained from National Institute on Aging (NIA) aged rodent colonies and C57BL/6J young adult mice (3–4 months old) were purchased from Jackson Laboratories. Upon arrival, the animals were maintained for at least 1 week to allow for habituation before experimentation. In experiments involving both sexes, mice were matched by sex and age. All animal procedures were approved by the Institutional Animal Care and Use Committee (IACUC) of the Johns Hopkins University School of Medicine and were conducted in accordance with ethical guidelines for animal research.

### 2.2 Anesthesia and surgery

A laparotomy was performed under isoflurane anesthesia with minor modifications from previous studies.^48^, ^49^ Anesthesia was induced and maintained at 2.1% isoflurane (IsoSol™, VEDCO) with an oxygen flow rate of 0.5–1 L/min using a rodent inhalation anesthesia apparatus (RC^2^ rodent circuit controller, cat # 922100, VetEquip^®^ inhalation anesthesia systems). Animals were initially placed in an induction chamber and then transferred to a nose cone. The surgical site was shaved, sterilely prepared with betadine scrub, and sterilely draped. A 1.5 cm incision was made in the middle line of the abdomen through the skin and muscle wall, and a sterile probe was inserted into the abdominal cavity to gently manipulate internal organs. The muscle wall was closed with sterile chromic gut sutures, and the skin was closed using silk thread sutures. Respiratory rhythm, frequency, and paw coloration were monitored throughout surgery. All surgeries (∼10–15 min) were performed by one person to minimize variability. Sham control mice (anesthesia‐only group) were exposed to isoflurane anesthesia for 15 min under identical conditions.

### 2.3 Behavioral tests

Behavioral tests were conducted on male and female mice housed on a reversed 12‐h light/dark cycle starting 2 days after abdominal surgery. All tests were conducted during the dark phase. Following 1 week of habituation in the reversed cycle, mice underwent behavior tests in the following order (two separate cohorts were prepared to ensure consistency in postoperative time within 7 days): first cohort—open field test, elevated plus maze test, novel object recognition test, novel location recognition test; second cohort—Barns maze test. The interval between behavioral tests was at least 24 hours. Each apparatus was cleaned with 70% ethanol between animals to eliminate odor cues.

#### 2.3.1 Open field test

The open field test was conducted following our previously published methods with minor modification.^50^, ^51^ Locomotor activity was assessed over a 30‐min period in a 40 × 40 cm activity chamber equipped with infrared beams (PAS system, San Diego Instruments Inc.). Horizontal and vertical locomotor activities in both the center and periphery of the chamber were automatically quantified infrared beam breaks.

#### 2.3.2 Elevated plus maze test

The elevated plus maze test was performed to assess anxiety‐like behaviors according to our published protocol.^50^, ^51^ Mice were placed at the center intersection of the elevated plus maze (San Diego Instruments, Inc.), and recorded for 5 minutes. The number of entries into the open and closed arms, as well as the time spent in each arm, were analyzed.

#### 2.3.3 Novel object recognition test and novel location recognition test

The novel object recognition test and novel location recognition test were conducted following our published methods with minor modification.^51^, ^52^ Mice were individually habituated for 3 consecutive days (10 min/day) in a Plexiglas open‐field arena (25 × 25 × 25 cm). In the training phase after habituation, each mouse was allowed to explore the arena with two identical objects positioned in adjacent corners of the floor for 5 minutes. About 30 minutes later, during the test phase, one object was replaced (novel object recognition test) or repositioned (novel location recognition test). The novel object was similar in size to the familiar object but different in shape and color. Exploration of the familiar versus novel object or position was recorded for 5 minutes, with recognition memory calculated as the proportion of time spent sniffing the novel relative to the total time spent sniffing both objects. Individual sniffing of the subject mice was manually scored from video recordings by researchers who were blinded to the group assignment.

#### 2.3.4 Barnes maze test

The Barnes maze consisted of a 92 cm diameter gray plastic circle elevated 105 cm with 20 evenly spaced holes (diameter: 5 cm, between holes: 7.5 cm) around its perimeter. Beneath a designated escape hole, a black plastic chamber could be inserted (target box), which allows the mouse to escape from the aversive stimuli (Bright light: 1100 lx) on circular plat form. During each training trial, mice were placed in the center of the maze for 10 seconds followed by exposure to the whole maze under bright light for 3 minutes, with the goal of entering the escape box. If a mouse fails to enter the escape box, the experimenter gently guided it into the escape box. Training was performed for 4 consecutive days (four trials per day). The target box location was kept consistent per animal. During the test session, 24 hours after the last training date, the mice explored the target box in the maze under bright light. The time spent finding and entering the target box was recorded using ANY‐maze tracking system (Stoelting Co.).

### 2.4 Intra cisterna magna tracer injection

Following anesthetization, animals were placed in a stereotaxic frame and the midline incision was made to expose the atlantooccipital membrane. A 30‐GA needle attached to a 100 µL HAMILTON syringe (cat # 8120, HAMILTON) with 0.011‐inch tube (cat # 22‐204008, fisher scientific) was inserted under visual guidance into cisterna magna to a depth of 1–1.5 mm, enduring that the needle tip entered the open subarachnoid space. The fluorescent CSF tracer (Albumin from bovine serum‐Alexa Fluor 647 conjugate, cat # A34785, Dextran‐Fluorescein‐3 kDa, cat # D3305, ThermoFisher scientific) was prepared in artificial CSF (aCSF) at a concentration of 1% (w/v). A total of 10 µL of CSF tracer was injected at a rate of 1 µL/min over a 10 min period. To visualize tracer penetration from the subarachnoid space into the brain parenchyma, mouse brains were extracted 30 min after intracisternal tracer injection and fixed by immersion for 24 hours in 4% paraformaldehyde (PFA). Coronal sections were obtained at 100 µm with a cryostat (cat # CM 3050S, Leica). Tracer distribution in the brain was acquired on whole‐slice brain sections using MetaXpress 6.0 slide scanning (Molecular Devices). The signal intensity of tracer was measured with manually outlined for targeted brain regions using ImageJ‐FIJI software. Signal intensity was measured from two brain sections per mouse for each condition. Identical parameters for all imaging and threshold settings were used for all groups to minimize experimental bias.

### 2.5 Immunohistochemistry

Immunohistochemistry was performed using our previously published methods with some modifications.^51^, ^53^, ^54^ Mouse brains were extracted after perfusion with 4% PFA. The fixed brains were embedded in cryocompound (Sakura Finetek USA) after replacement of PFA with 20% and 30% sucrose in phosphate buffered saline (PBS). Coronal sections were obtained at 100 µm with a cryostat (cat # CM 3050S, Leica). The sections were heated in HistoVT One solution (cat # 06380‐05, Nacalai Tesque) for 30 minutes at 70°C for antigen retrieval. The sections were then washed with PBS containing 0.5% Triton X‐100, followed by blocking with 0.5% Triton X‐100 and 10% normal goat serum for 1 hour. After blocking, sections were incubated with primary antibody [chicken anti‐GFAP antibody (cat # ab4674, Abcam, 1:500), rabbit anti‐Aldh1l1 antibody (cat # 85828S, Cell Signaling Technology, 1:50), mouse anti‐Aldh1l1 antibody (cat # MABN495, Millipore, 1:100), mouse anti‐GCPII antibody (cat # NBP1‐45057, NOVUS, 1:200), rat anti‐GFP antibody (cat # 04404‐84, Nacalai, 1:500), rat anti‐laminin antibody (cat # MA1‐06100, Invitrogen, 1:50), and rabbit anti‐AQP4 antibody (cat # sc‐20812, Santa Cruz, 1:200), mouse anti‐AQP4 antibody (cat # ab9512, Abcam, 1:100)] at 4°C overnight. An additional incubation with secondary antibodies conjugated to Alexa 488 (cat # A‐21467, Invitrogen, 1:400), Alexa 568 (cat # A‐11011, Invitrogen, 1:400), and Alexa 647 (cat # A‐21247, Invitrogen, 1:400) was performed for 2 hours. Nuclei were labeled with DAPI (4′,6‐diamidino‐2‐phenylindole; cat # 10236276001, Roche).

### 2.6 Image analysis

Immunofluorescence images were acquired using the Zeiss LSM700 confocal microscope with ZEN 2010 software (Carl Zeiss). The hippocampal CA1 was defined as anteroposterior (AP): −1.22 to −2.06 mm, mediolateral (ML): ± 1.6 mm from bregma, dorsoventral (DV): −1.05 to −1.2 mm from the dura according to the brain atlas. For fluorescence measurements, sections were imaged using a 40× oil immersion objective lens to collect *z*‐stack images of 25 optical sections at a step size of 1‐µm thickness under identical imaging settings across groups. Quantitative image analysis was performed using ImageJ‐FIJI software. For GFAP intensity analysis, perivascular regions were defined as areas surrounding Laminin^+^ blood vessels, and mean GFAP fluorescence intensity within these regions was measured as an index of perivascular GFAP immunoreactivity, with reference to previous study.^55^ For AQP4 intensity analysis, GFAP^+^ regions overlapping with Laminin^+^ blood vessels were defined as vessel‐associated perivascular astrocytic regions, whereas GFAP^+^/Laminin^−^ regions were classified as non–vessel‐associated astrocytic areas. Mean AQP4 fluorescence intensity was measured within each region based on a previously described approach.^56^ For GCPII knockdown analysis, enhanced green fluorescent protein (EGFP)^+^ astrocytic regions were defined by the overlap of EGFP and astrocyte marker (GFAP and Aldh1l1) signals, independently of the GCPII channel. Thresholded binary masks of these regions were applied to the corresponding GCPII channel images, and mean fluorescence intensity within the masked regions was measured. Signal intensity was measured and averaged from two brain sections per mouse for each condition. Identical imaging parameters and threshold settings were applied across all groups to minimize experimental bias. All measurements were performed by experimenters blinded to the experimental groups. High‐magnification confocal z‐stack images were acquired using a 63× oil‐immersion objective to examine AQP4 localization relative to blood vessels and perivascular astrocytic structures. Each z‐stack consisted of 25 optical sections acquired at 0.5‐µm intervals. Three‐dimensional rendering was performed using Imaris 11.0.0 software (Bitplane/Oxford Instruments). Blood vessel and astrocyte signals were segmented and rendered as semi‐transparent surface objects, and the unsegmented AQP4 fluorescence signal was overlaid onto these reconstructed structures to visualize its spatial relationship with the vessel surface and astrocytic processes.

### 2.7 Astrocyte‐specific single‐cell RNA sequencing (scRNA‐seq)

The astrocyte (ACSA‐2^+^) was collected from the hippocampus of aged mice 3 days after surgery using fluorescence‐activated cell sorting (FACS), following published methods.^51^ Briefly, mice were anesthetized and transcardially perfused with 20 mL of ice‐cold Hanks’ balanced salt solution (HBSS; cat # 55021C, Sigma‐Aldrich). Hippocampal tissues were dissected, minced in Hibernate A (cat # NC0442869, Fisher Scientific), and dissociated with the neural tissue dissociation kit (cat# 130‐093‐231, MACS Miltenyi Biotec). The homogenates were passed through a 70‐µm cell strainer and centrifuged at 300 g for 10 minutes. Supernatants were removed and cell pellets were resuspended and subjected to debris removal using the Miltenyi debris removal reagent (cat # 130‐109‐398, Miltenyi Biotec Inc) according to the manufacturer's instructions. Cell pellets were then resuspended in 50 µL of FACS buffer (0.5% bovine serum albumin in PBS). To block nonspecific binding, cells were incubated with anti‐CD16/CD32 antibody (cat # 101320, BioLegend, clone 93, 5 ng/µL) for 10 minutes at 4 °C. Cells were subsequently stained for 30 minutes at 4 °C with PE conjugated anti‐mouse ACSA‐2 (cat # 130‐123‐284, Miltenyi Biotec, clone IH3‐18A3, 1:25 dilution), LIVE/DEAD Fixable green dead cell Staining Solution (cat # L34970, Invitrogen), and Hoechst 33342 (cat # H3570, Invitrogen). After staining, cells were washed and resuspended in 300 µL of FACS buffer. Following staining, cells were fixed using the fixation buffer provided in the 10× Genomics GEM‐X Flex Sample Preparation v2 Kit (cat # 1000781, 10× Genomics), and incubated overnight at 4 °C. On the following day, ACSA‐2^+^ astrocytes were sorted using a FACS Aria Flow Cytometer. Collected cells were processed for long‐term storage following the GEM‐X Flex protocol CG000782, Rev C (10× Genomics) and stored at −80°C until all samples were collected for simultaneous library preparation to minimize batch effects. These cells were then transferred to the Johns Hopkins Single Cell & Transcriptomics core for subsequent processing. At the core facility, samples were processed in accordance with the post‐storage processing (CG000782 RevC) before counting using acridine orange and propidium iodide (AO/PI) on the Cellometer Ascend (Revvity Biosciences). Protocol CG000787 (Rev A, 10× Genomics) was followed to generate the three four‐plex GEM‐X flex libraries. Libraries were quantified on a Fragment Analyzer (Agilent Biosciences), before sequencing on a NovaSeqX with 10% PhiX (one‐fourth of a 10B flow cell lane per four‐plex library, three‐fourth of a lane total).

### 2.8 scRNA‐seq data analysis

Raw sequencing data were preprocessed with cellranger‐9.0.1 (10× Genomics), in which reads were aligned to the GRCm39 mouse reference genome. Expression data were processed with Scanpy^57^ (ver. 1.10.3) in Python (ver. 3.13.7). We removed doublets using Scrublet^58^ (ver. 0.2.3). We then removed cells with fewer than 500 genes detected, fewer than 1,000 total unique molecular identifiers (UMIs), greater than 10% of reads mapping to mitochondrial genes, and greater than 5% of reads mapping to hemoglobin genes. Genes detected in fewer than 10 cells were removed. Read counts were normalized to counts per million (CPM) and log‐transformed (log(CPM+1)). Highly variable genes (HVGs) were identified using Scanpy's sc.pp.highly_variable_genes function with parameters min_mean = 0.005, max_mean = 8, and min_disp = 0.1, yielding 3,777 HVGs. Gene expression values were then scaled with a maximum value of 10. We performed dimensionality reduction using principal component analysis (PCA) with the “arpack” solver on the HVGs. To correct for batch effects, we applied Harmony^59^ integration on the PCA embeddings using Harmonypy (ver. 0.0.10). A nearest neighbor graph was constructed using the batch‐corrected Harmony coordinates (50 neighbors, 20 PCs), followed by uniform manifold approximation and projection (UMAP) dimensionality reduction for visualization. Astrocytes (*n* = 9,574 cells) were grouped into 11 different sub‐clusters using the Leiden algorithm.^60^ Cluster marker genes were identified using sc.tl.rank_genes_groups from Scanpy (Wilcoxon rank‐sum test, adjusted *p* < 0.05; log2FC > 1). Astrocyte sub‐cluster distribution across samples was analyzed using scCODA^61^ (ver. 0.1.9). Astrocyte sub‐cluster‐specific pseudobulk differential gene expression analysis was performed using DESeq2^62^ (ver. 1.50.2) for sub‐clusters 0–7 using batch as a covariate. Sub‐clusters 8–10 were removed from the analysis due to their small number of cells. Chromosome Y genes were also removed from the analysis. Pseudobulk expression profiles were generated by aggregating counts across cells for each sample‐cluster combination using aggregateAcrossCells from scuttle^63^ (ver. 1.20.0). Gene set enrichment analysis (GSEA)^64^ was performed with fgsea (ver. 1.36.0) using sign(log2FC) × ‐log10(p‐value) as the ranking metric. Targeted GSEA was conducted on gene ontology biological processes (GO:BP) terms filtered for well‐established astrocyte‐related amino acids (glutamate, glutamine, gamma‐aminoburyric acid [GABA], aspartate, glycine, and serine).

### 2.9 Measurement of glutamate content in the hippocampus

Glutamate concentration in the hippocampal tissue was measured using the colorimetric glutamate assay kit (cat # ab83389, Abcam). At 2 days after surgery, mice were anesthetized and transcardially perfused with 20 mL cold PBS. Both hippocampi were isolated, washed in cold PBS, and homogenized in 500 µL of Assay Buffer according to manufacturer instructions. After cell lysate was incubated with the reaction buffer at 37°C for 30 min protected from light, glutamate concentration was quantified at the wavelength of 450 nm by SpectraMax i3X (Molecular Devices).

### 2.10 Human data collection and expression analysis

Transcriptomic data from human *post mortem* brains were obtained from the publicly available Brainseq2 dataset (https://eqtl.brainseq.org/phase2/).^65^ The relationships between *FOLH1, GLS, SLC1A3, SLC1A2, SLC7A11* expression and age were analyzed in hippocampus and dorsolateral prefrontal cortex. For these analyses, we used a linear model with gene expression (RPKM) as dependent variable, and Age as predictor, with sex, race, and RNA integrity number as covariates. Sex‐stratified analyses were also performed, and Spearman's rank correlation coefficients (rho) and p values were calculated separately for male and female samples to assess the relationship between adjusted gene expression and age.

### 2.11 RNA isolation and quantitative real‐time PCR (qPCR)

Total RNA was isolated from hippocampal tissue using the RNeasy Mini Kit (QIAGEN). qPCR was performed using the TaqMan system (Applied Biosystems). Briefly, cDNA was synthesized from total RNA (10–100 ng) using the SuperScript® III CellsDirect cDNA Synthesis Kit (Life Technologies). The qPCR reactions contained diluted cDNA from the synthesis reaction and 200 nM of forward and reverse TaqMan primers specific to the genes of interest (Assay ID for Folh1: Mm00489655_m1, Applied Biosystems). GAPDH primers (Assay ID: Mm99999915_g1, Applied Biosystems) were used as a normalization control. The qPCR reaction was run on Applied Biosystems QuantStudio™ 5 under the following conditions: 50°C, 2 minutes; 95°C, 2 minutes; 60 cycles of 95°C, 1 second; and 60°C, 20 seconds.

### 2.12 CD11b^+^ cells and ACSA‐2^+^ cells isolation

ACSA‐2^+^ astrocytes and microglia‐enriched CD11b^+^ cells were separately isolated from the same aged mouse hippocampus according to our published protocol with minor modifications.^51^ Briefly, mice were anesthetized and transcardially perfused with 20 mL cold PBS. The hippocampal tissues were isolated, cut into small pieces in HBSS (cat # 55021C, Sigma‐Aldrich), and dissociated with the neural tissue dissociation kit (For astrocyte: cat# 130‐093‐231 and for microglia: cat # 130‐092‐628, MACS Miltenyi Biotec). After passing through a 70‐µm cell strainer, homogenates were centrifuged at 300 g for 10 minutes. Supernatants were removed and cell pellets were resuspended. Myelin was removed by Myelin Removal Beads II (cat # 130‐096‐733, MACS Miltenyi Biotec). Myelin‐removed cell pellets were resuspended and incubated with ACSA‐2 MicroBeads (cat # 130‐097‐678, MACS Miltenyi Biotec) or CD11b MicroBeads (cat # 130‐049‐601, MACS Miltenyi Biotec) for 15 minutes, then loaded onto LS columns and separated on a quadroMACS magnet. ACSA‐2^+^ cells or CD11b^+^ cells were flushed out from the LS columns, and the flow‐through fraction, representing ACSA‐2‐negative remaining cells, was collected and used for subsequent qPCR analysis.

### 2.13 GCPII enzyme activity measurement

To confirm GCPII target engagement, an enzymatic activity assay was completed on isolated ACSA‐2^+^ astrocytes and microglia‐enriched CD11b^+^ cells based on previously described methods.^66^, ^67^ Astrocyte and microglia pellets were suspended in 150 µL ice‐cold Tris buffer (40 mM, pH 7.5) containing protease inhibitors (Roche, Complete Protease Inhibitor Cocktail, 1 tablet in 5 mL, cat # 50‐100‐3301, Fisher Scientific) and then sonicated using Kontes' Micro Ultrasonic Cell Disrupter (three 10‐second pulses, 30 seconds between pulses). The homogenates were spun down at 16,000 × *g* for 2 minutes at 4°C, and the supernatants were collected for GCPII activity and total protein quantification with BioRad's Detergent Compatible Protein Assay kit (Cat # 5000113,5000114, BioRad). The assay mix containing 1 mM cobalt chloride and 40 nM NAA‐[3H]‐G was loaded into a 96‐well plate (25 µL/well) and warmed for 5 minutes at 37°C. After this pre‐warming step, 25 µL of the astrocyte lysates were added to the plate in triplicate, for a total reaction volume of 50 µL/well. The plates were incubated at 37°C for 3 hours with gentle shaking. The reactions were stopped with 50 µL of ice‐cold sodium phosphate buffer containing 1‐mM EDTA (pH 7.4, 0.1 M). A 90‐µL aliquot from each well was transferred to 96‐well spin columns containing AG1 × 8 ion‐exchange resin and centrifuged at 1000 rpm for 7 min using a Beckman GS‐6R centrifuge equipped with a PTS‐2000 rotor. Columns were washed twice with 90 µL of 1‐M formic acid to ensure all [3H]‐G was eluted. A 200‐µL aliquot of the flow through from each well was transferred to a solid scintillator‐coated 96‐well plate (cat #: 6006633, Packard) and dried. The radioactivity corresponding to [3H]‐G was determined with a scintillation counter (Topcount NXT, Packard, counting efficiency 80%), and results were reported as fmol/mg/h.

### 2.14 Folh1 (GCPII) short hairpin RNA (shRNA) AAV viral production

To selectively knockdown *Folh1* (GCPII) expression in the astrocyte, we generated an astrocyte‐specific adeno‐associated viral (AAV) vector. Astrocyte‐specific shRNA constructs were designed and cloned by VectorBuilder using pAAV[miR30]‐GFAP‐EGFP‐shRNA‐WPRE constructs. Two shRNA target sequences were used for mouse Folh1 knockdown.

- mFolh1; 5′‐GGCCTGGATTTGGTTGAGTTAT‐3′, a sequence designed by VectorBuilder.
- mFolh1‐3; 5′‐CAGTGAGAGACTCCAGGACTT‐3′, a previously reported sequence.^68^

A scrambled shRNA sequence with no homology to any known mouse mRNAs (5′‐ACCTAAGGTTAAGTCGCCCTCG‐3′) was used as a negative control. All AAV vectors (serotype 5) were packaged and purified by VectorBuilder.

### 2.15 Stereotaxic viral injection

Stereotaxic AAV injections were performed 3 weeks prior to abdominal surgery as previously described.^52^ Mice were anesthetized with an intraperitoneal administration of a mixed solution containing ketamine (100 mg/kg), xylazine (7.5 mg/kg), and buprenorphine (0.05 mg/kg).^53^ After securing the mouse in a stereotaxic frame, a small craniotomy was performed to expose the skull, allowing a small hole to be drilled at the appropriate coordinates. A total of 500 nL AAV was injected into the hippocampus using a 10 µL syringe (Hamilton, USA) at a rate of 50 nL/min. Following injection, the needle was left in place for an additional 5 minutes before being slowly withdrawn. The incision was sutured, and mice were returned to their home cages and allowed to recover for at least 3 weeks before subsequent abdominal surgery. AAV was injected bilaterally at four sites within the hippocampal region using the following stereotaxic coordinates (relative to bregma):

Site 1: AP: –1.8 mm, ML: ± 1.0 mm, DV: –1.7 mm; Site 2: AP: –2.5 mm, ML: ± 2.0 mm, DV: –1.8 mm.

### 2.16 GCPII inhibitor (2‐PMPA) treatment

The 2‐PMPA (2‐(phosphonomethyl)‐pentanedioic acid) was prepared as previously described.^69^ Based on our published pharmacokinetic and efficacy studies,^70^ 2‐PMPA was intraperitoneally (i.p.) administered at a dose of 100 mg/kg in HEPES‐buffered saline (cat # H‐2430‐500ML, AG Scientific). Aged mice were treated with 2‐PMPA or vehicle 30 minutes before abdominal surgery. After surgery, the mice were daily treated 2‐PMPA or vehicle until the end of behavioral testing and brain extraction. Throughout all procedures, animals were handled gently to minimize stress and reduce potential confounding effect on the experimental outcomes.

### 2.17 Statistical analyses

Behavioral and biochemical data were analyzed by one‐way analysis of variance (ANOVA) or two‐way mixed ANOVA to identify each effect with the group (surgery‐exposed mice and sham control mice) and treatment (knockdown virus or 2‐PMPA and vehicle) as independent factors. Tukey's post‐hoc test was used to determine statistically significant differences between groups. Two‐tailed analyses of unpaired Student's *t*‐tests were also used for data analysis in comparing surgery‐exposed mice and littermate sham control mice. These statistical analyses were conducted by using GraphPad Prism 10 (GraphPad Software). A value of *p *< 0.05 was considered statistically significant. All data are presented as means ± standard error of the mean (s.e.m.).

## 3 RESULTS

### 3.1 Abdominal surgery induces deficits in recognition and spatial memory and impairs glymphatic influx in the hippocampus in aged male mice

**FIGURE 1:**
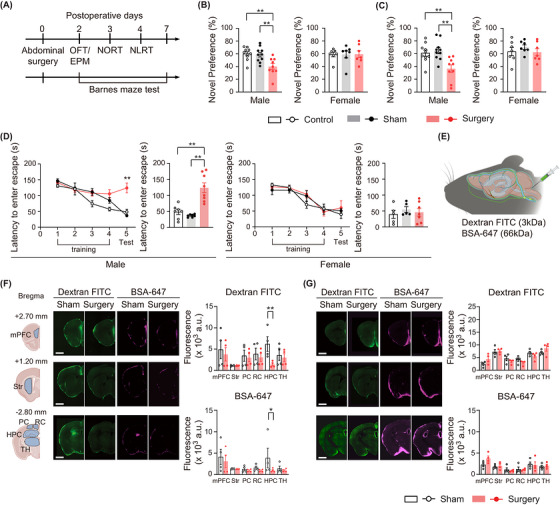
Abdominal surgery induces male‐specific deficits in memory function and hippocampal glymphatic influx in aged mice. (A) Schematic diagram of the experimental timeline. Mice underwent abdominal surgery on post‐operative day 0. Behavioral analysis was performed using two separate cohorts: one cohort was used for open field test (OFT), elevated plus maze test (EPM), novel object recognition test (NORT), and novel location recognition test (NLRT), and another independent cohort was used for the Barnes maze test. (B) Preference for the novel object in the novel object recognition test for male (control, *n* = 10; sham, *n* = 10; surgery, *n* = 9) and female (control, *n* = 7; sham, *n* = 7; surgery, *n* = 7) mice. (C) Preference for the novel location in the novel location recognition test for male (control, *n* = 10; sham, *n* = 10; surgery, *n* = 9) and female (control, *n* = 7; sham, *n* = 7; surgery, *n* = 7) mice. (D) Latency to enter the escape box during the training and test phases of the Barnes maze test for male (control, *n* = 7; sham, *n* = 7; surgery, *n* = 8) and female (control, *n* = 5; sham, *n* = 5; surgery, *n* = 7) mice. (B, C, D) *p < 0.05, **p < 0.01, ***p < 0.001, determined by one‐way analysis of variance (ANOVA) followed by Tukey's post hoc test. (E) Schematic of intracisternal magna (i.c.m.) injection performed 3 days after surgery. (F) Left, schematic illustrating brain regions analyzed for tracer fluorescent signal: medial prefrontal cortex (mPFC), parietal cortex (PC), retrosplenial cortex (RC), hippocampus (HPC), thalamus (TH), and striatum (Str). Middle, representative images showing tracer distribution (green, Dextran‐fluorescein isothiocyanate [FITC]; magenta, BSA‐647) in male mice; scale bars, 500 µm. Right, quantification of tracer penetration in indicated brain regions (*n* = 4 mice per group). *p < 0.05, **p < 0.01, determined by unpaired two‐tailed Student's *t*‐test. (G) Left, representative images showing tracer distribution (green, Dextran‐FITC; magenta, BSA‐647) in female mice; scale bars, 500 µm. Right, quantification of tracer penetration in indicated brain regions (*n* = 4 mice per group). Data represent mean ± SEM.

To evaluate the effects of abdominal surgery on cognitive function, we evaluated behavioral outcomes in 18‐ to 20‐month‐old male and female mice subjected to inhalation anesthesia (2.1% isoflurane) and subsequent abdominal surgery following two days of post‐operative recovery (Figure 1A). Previous studies have reported surgery‐induced recognition memory deficits in aged male rats.^18^ Consistent with these findings, we observed deficits in recognition memory in surgery‐exposed male mice, compared to male mice underwent inhalation anesthesia without abdominal surgery (Sham) and naïve control male mice (Control), as assessed by the novel object recognition test and novel location recognition test (Figure 1B, C). We also examined spatial learning and memory using the Barnes maze test. During the training (acquisition) phase, all groups showed progressively reduced escape latency (Figure 1D). However, during the retention test phase, surgery‐exposed male mice exhibited prolonged escape latency, suggesting impaired memory recall (Figure 1D). To exclude potential confounding factors, we assessed locomotor activity and anxiety‐like behaviors using the open field test and elevated plus maze test. Neither anesthesia nor surgery affected these measures in male or female mice, confirming that cognitive deficits were not due to changes in general activity or anxiety levels (Figure S1A,B). Interestingly, female mice did not exhibit surgery‐induced deficits in recognition and spatial memory, suggesting that sex‐dependent differences may influence susceptibility to cognitive deficits in this abdominal surgery model (Figure 1B‐D).

Glymphatic dysfunction has been increasingly implicated in age‐related cognitive decline.^55^ To assess whether abdominal surgery further impairs glymphatic clearance in aged mice, we injected the fluorescent tracers (BSA‐647; molecular weight 66 kDa and fluorescein isothiocyanate (FITC)–dextran; molecular weight 3 kDa) into the cisterna magna, a subarachnoid space facilitating CSF flow, in aged mice 3 days after surgery (Figure 1E). AtL 30 minutes after tracer infusion, tracer penetration was evaluated by measurement of the fluorescence signals. In aged male mice, we observed reduced penetration of both tracers in the hippocampus following surgery, whereas no changes were detected in other brain regions involved in recognition and spatial memory, including the medial prefrontal cortex, parietal cortex, retrosplenial cortex, thalamus, and striatum (Figure 1F). In contrast, abdominal surgery did not alter tracer influx in aged female mice (Figure 1G). These findings suggest that abdominal surgery impairs glymphatic influx selectively in aged males.

### 3.2 Abdominal surgery changes perivascular astrocyte reactivity, impairs AQP4 perivascular polarity, and alters glutamate signaling pathways in the hippocampus of aged male mice

**FIGURE 2:**
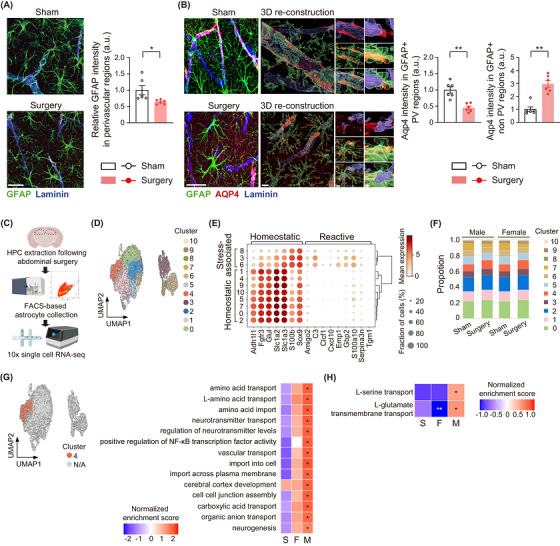
Abdominal surgery induces altered perivascular astrocyte reactivity, loss of aquaporin‐4 (AQP4) polarization, and male‐specific alteration of glutamate signaling pathways in the hippocampal astrocytes of aged mice. (A) Immunohistochemistry for glial fibrillary acidic protein (GFAP; green) and Laminin (blue) in hippocampal CA1 region 3 days after surgery; scale bars, 20 µm. Right, quantification of relative GFAP fluorescence intensity in perivascular regions surrounding Laminin^+^ vessels (*n* = 6 slices from three mice per group). (B) Left, representative immunohistochemical images for GFAP (green), AQP4 (red), and Laminin (blue) in hippocampal CA1 region; scale bar, 20 µm. Middle, 3D‐rendered reconstructions and high‐magnification images showing AQP4 localization relative to Laminin^+^ vessels and perivascular (PV) astrocytic structures; scale bar, 10 and 4 µm. Right, quantification of AQP4 signal intensity in GFAP^+^ PV regions and GFAP^+^ non‐PV regions (*n* = 6 slices from three mice per group). (A, B) **p < 0.01, ***p < 0.001, determined by unpaired two‐tailed Student's *t*‐test. Data represent mean ± SEM. (C) Experimental flow of fluorescence‐activated cell sorting (FACS)‐based hippocampal astrocyte isolation by using a marker (ACSA‐2) 3 days after surgery, followed by single cell RNA sequencing (scRNA‐seq). (D) Uniform manifold approximation and projection (UMAP) clustering of scRNA‐seq data from hippocampal astrocytes (total 9,574 astrocytes) across female‐sham, female‐surgery, male‐sham, and male‐surgery conditions (*n* = 3 mice per group). (E) Dot plot showing the expression of homeostatic and reactive astrocyte state marker genes across identified clusters. (F) Proportional distribution of astrocyte clusters across the four conditions. (G) Left, UMAP plot highlighting astrocyte cluster 4 (total 907 astrocytes). Right, gene set enrichment analysis (GSEA) of cluster 4 showing enriched gene ontology (GO) biological process pathways across the three comparisons (female‐sham vs. male‐sham; S, female‐sham vs. female‐surgery, F; and male‐sham vs. male‐surgery, M), showing normalized enrichment score (NES) values. (H) GSEA heatmap of amino acid–related GO biological process pathways in astrocyte cluster 4 across the three group comparisons, showing NES values. (G, H) *p < 0.1, **p < 0.05, false discovery rate (FDR)‐adjusted *p*‐values.

Having shown that the glymphatic system is modulated by perivascular astrocytes and their AQP4‐enriched endfeet, which facilitate CSF and interstitial fluid exchange,^36^, ^40^, ^41^ we next investigated whether abdominal surgery alters perivascular astrocytes and their AQP4 localization. We observed reduction in GFAP immunoreactivity in perivascular regions of the hippocampus in surgery‐exposed male mice (Figure 2A). To assess surgery‐induced changes in AQP4 distribution within perivascular astrocytes, we performed co‐immunostaining for AQP4, GFAP, and Aldh1l1 (astrocyte markers), and laminin (vascular marker). High‐magnification confocal z‐stack imaging and 3D rendering confirmed that AQP4 immunoreactivity was localized within GFAP‐positive perivascular astrocytic regions, and colocalization with Aldh1l1‐positive astrocytes further confirmed its astrocytic identity (Figure 2B and Figure S2A). In surgery‐exposed mice, AQP4 immunoreactivity was reduced in perivascular regions, whereas it was increased in non‐perivascular astrocytic regions (Figure 2B). These results indicate that abdominal surgery disrupts the perivascular polarity of AQP4.

To define sex‐specific and astrocyte‐dependent molecular responses to abdominal surgery, we performed scRNA‐seq on FACS‐isolated hippocampal astrocytes from surgery‐exposed and sham control mice of both sexes (Figure 2C). We identified 11 transcriptionally distinct astrocyte states, comprising both homeostatic and stress‐associated populations (Figure 2D, E and Figure S2B,C). Canonical homeostatic astrocyte genes (e.g., *Slc1a2*, *Slc1a3*, *Glul*, and *Aldh1l1*) were enriched in clusters 0–2, 4, 5, 7, and 9–10, whereas reactivity‐associated astrocyte genes (e.g., *C3*, *Gbp2*, and *S100a10*) predominated in clusters 3, 6, and 8 (Figure 2E and Figure S2B). Cluster distribution analysis revealed that astrocyte state composition was broadly conserved between sexes and remained largely unchanged following surgery (Figure 2F and Figure S2C). Differential expression analysis identified a limited number of differentially expressed genes across sex and surgical conditions (Table S1). These results suggest that abdominal surgery may induce sex‐specific coordinated transcriptional alterations at the pathway level, rather than robust changes in individual genes. To identify such coordinated changes, we performed cluster‐specific GSEA for three pairwise comparisons: sham‐male versus sham‐female, sham‐male versus surgery‐male, and sham‐female versus surgery‐female. Each comparison revealed significant pathway alterations across multiple astrocyte clusters (Tables S2–S4). Notably, cluster 4 emerged as the only astrocyte population showing significant pathway enrichment specifically in the sham‐male versus surgery‐male comparison (Tables S2–S4). The enriched pathways converged on amino acid and neurotransmitter regulation (Figure 2G). To further delineate the molecular basis of this sex‐specific response, we performed targeted GSEA focusing on gene ontology terms related to key astrocyte‐associated amino acids. This analysis demonstrated selective enrichment of L‐glutamate‐related signaling in the sham‐male versus surgery‐male comparison (Figure 2H). These findings highlight a male‐specific enhancement of glutamate signaling pathways in a distinct astrocyte subpopulation following abdominal surgery.

### 3.3 Abdominal surgery induces male‐specific glutamate elevation in the hippocampus and upregulates GCPII activity in hippocampal astrocytes

**FIGURE 3:**
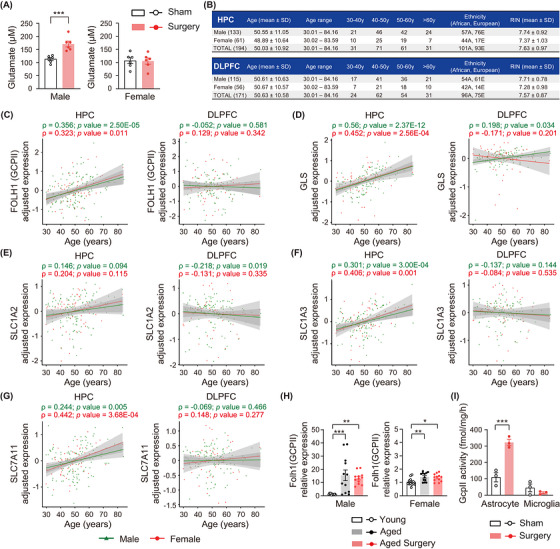
Elevation of hippocampal glutamate and increased astrocyte glutamate carboxypeptidase II (GCPII) activity in aged male mice following abdominal surgery. (A) Glutamate concentration in the hippocampus of male and female mice measured 3 days after surgery (*n* = 6 per group). ***p < 0.001, determined by unpaired two‐tailed Student's *t*‐test. (B) Demographics of publicly available bulk RNA‐seq datasets of human *post mortem* brains. Characteristics of the neurotypical controls samples used for the analyses of the relationship between age and *FOLH1*, *GLS*, *SLC1A2*, *SLC1A3*, and *SLC7A11* expression in the hippocampus (top) and the dorsolateral prefrontal cortex (bottom) are shown. (C–G) Scatterplots of adjusted gene expression level (RPKM, reads per kilobase per million mapped reads, adjusted by race and RNA Integrity Number) for *FOLH1* (GCPII), *GLS*, *SLC1A2*, *SLC1A3*, and *SLC7A11* in human *post mortem* hippocampus (HPC; left panels, *N* = 194) and dorsolateral prefrontal cortex (DLPFC; right panels, *N* = 171), stratified by sex (male, green; female, red). Lines represent linear regression fits and Spearman's correlation coefficients ρ and *p* values are shown above each plot for males and females. (H) Relative *Folh1*(GCPII) mRNA expression in hippocampus of male and female mice across young, aged, and aged surgery groups (*n* = 12 per group). (I) GCPII activity in hippocampal ACSA‐2^+^ astrocytes and microglia‐enriched CD11b^+^ cells (*n* = 3 per group, pooled hippocampus from two mice per sample). (H, I) *p < 0.05, **p < 0.01, ***p < 0.001, determined by two‐way analysis of variance (ANOVA) with Tukey's post hoc test. Data represent mean ± SEM.

Given that excess glutamate impairs hippocampal‐dependent memory function,^71^, ^72^ we investigated whether glutamate dysregulation serves as biochemical substrate for surgery‐induced memory deficits. We found an increase in total glutamate content in the hippocampus of surgery‐exposed male mice following postoperative recovery, whereas no such change was observed in surgery‐exposed female mice (Figure 3A). These findings, together with our immunohistochemical analyses of astrocytes (Figure 2A,B), are consistent with previous in vitro studies demonstrating that excess glutamate can alter AQP4 expression.^42^ Our results indicate a male‐specific glutamate dysregulation in response to abdominal surgery, potentially contributing to sex‐dependent vulnerability to cognitive deficits.

Glutamate homeostasis in the brain is regulated by multiple astrocytic molecules, including glutamate‐producing enzymes such as GCPII and glutaminase (GLS), as well as glutamate transporters such as GLT‐1, GLAST, and the light chain subunit of the cystine/glutamate antiporter system Xc^−^ (xCT).^31^, ^46^, ^73^ Age‐related alterations in the expression of these molecules may increase vulnerability to surgery‐induced disruptions in glutamate homeostasis. Supporting this possibility, our bulk RNA sequencing analysis of *post mortem* hippocampal tissue from human healthy individuals (*N* = 194; age range from 30 to 84 years; Figure 3B) revealed age‐associated increases in the expression of *FOLH1, GLS*, *SLC1A2, SLC1A3, SLC7A11* (encoding GCPII, GLS, GLT‐1, GLAST, and xCT, respectively), whereas no such age‐related changes were observed in the prefrontal cortex (*N* = 171) (Figure 3C‐G).

Among these age‐associated genes, we focused on *FOLH1* (GCPII), because GCPII is highly expressed in astrocytes and uniquely generates extracellular glutamate through the hydrolysis of N‐acetylaspartylglutamate (NAAG).^46^, ^74^, ^75^, ^76^, ^77^ Sex‐stratified analysis demonstrated significant positive age correlations for FOLH1 expression in both males (rho = 0.356, *p* = 2.5E‐05) and females (rho = 0.323, *p* = 0.011) (Figure 3C). GCPII may represent an upstream factor contributing to age‐related vulnerability to glutamate dysregulation and PND pathophysiology. We observed that hippocampal *Folh1* mRNA expression was higher in aged male mice than in young male mice, with an approximately 14‐fold increase, whereas in females the increase was limited to approximately 1.4‐fold (Figure 3H). Interestingly, abdominal surgery did not alter *Folh1* expression in aged mice (Figure 3H), suggesting that abdominal surgery may instead influence GCPII enzymatic activity. To test this, we measured GCPII enzymatic activity in astrocyte‐enriched ACSA‐2^+^ cells isolated from the hippocampus and found that GCPII activity was upregulated in surgery‐exposed male mice (Figure 3I). In contrast, GCPII activity in microglia‐enriched CD11b^+^ cells was substantially lower than in astrocyte‐enriched ACSA‐2^+^ cells in sham control mice and was unaffected by surgery (Figure 3I). These findings suggest that abdominal surgery selectively enhances GCPII activity in astrocytes, which may promote excess glutamate accumulation in the hippocampus and contribute to cognitive deficits in aged male mice.

### 3.4 Astrocyte‐specific suppression of GCPII and pharmacological inhibition of GCPII alleviate phenotypes induced by abdominal surgery in aged male mice

**FIGURE 4:**
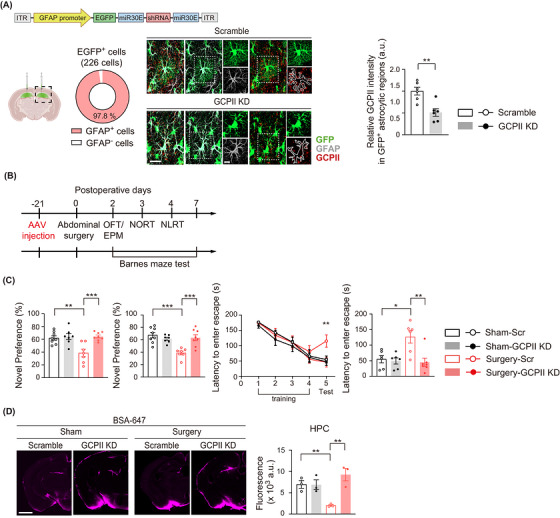
Astrocyte‐specific suppression of glutamate carboxypeptidase II (GCPII) alleviate cognitive impairments and hippocampal glymphatic influx in aged male mice following abdominal surgery. (A) Schematic illustrating the experimental approach. Top and left, an astrocyte‐specific AAV5‐GFAP‐EGFP‐[miR30]‐shRNA vector targeting Folh1 (GCPII) was bilaterally injected into the hippocampus. Quantification of adeno‐associated viral (AAV) ‐infected cells showing that the majority of enhanced green fluorescent protein (EGFP) ‐positive cells were glial fibrillary acidic protein (GFAP)+ cells. Middle, representative images of the hippocampal CA1 region from scramble and GCPII KD mice. Images are shown as GFP (green)/GFAP (gray)/GCPII (red), green fluorescent protein (GFP)/GFAP, and GFP/GCPII channels from left to right, with high‐magnification views highlighting individual EGFP‐positive astrocytes; scale bars, 20 and 10 µm. Right, quantification of relative GCPII fluorescence intensity in GFP+ astrocytic regions. (*n* = 6 slices from three mice per group). **p < 0.01, determined by unpaired two‐tailed Student's *t*‐test. (B) Schematic diagram of the experimental timeline. AAV was administered 21 days prior to abdominal surgery. Behavioral analysis was performed using two separate cohorts: one cohort was used for open field test (OFT), elevated plus maze test (EPM), novel object recognition test (NORT), and novel location recognition test (NLRT), and another independent cohort was used for the Barnes maze test during post‐operative days 2–7. (C) Left, preference for the novel object in the novel object recognition test, Middle, preference for the novel location recognition test (Sham‐Scr, *n* = 8; Sham‐GCPII KD, *n* = 7; Surgery‐Scr, *n* = 7; Surgery‐GCPII KD, *n* = 8). Right, latency to enter the escape box during the training and test phases of the Barnes maze test for sham and surgery groups treated with scrambled control (Scr) or shRNA knockdown of GCPII (GCPII KD) (Sham‐Scr, *n* = 6; Sham‐GCPII KD, *n* = 6; Surgery‐Scr, *n* = 6; Surgery‐GCPII KD, *n* = 7). (D) Left, representative images showing tracer distribution (magenta, BSA‐647) in in sham and surgery groups treated with scrambled control (Scr) or shRNA for knockdown of GCPII (GCPII KD); scale bars, 500 µm. Right, quantification of tracer penetration in the hippocampus (*n* = 3 mice per group). (C, D) *p < 0.05, **p < 0.01, ***p < 0.001, determined two‐way analysis of variance (ANOVA) with Tukey's post hoc test. Data represent mean ± SEM.

Our results above indicate that abdominal surgery impairs glymphatic influx, potentially mediated by loss of perivascular AQP4 polarization in astrocytes. This process may be driven by upregulation of GCPII activity and excess glutamate production in the hippocampus, leading to deficits in recognition and spatial memory. Given the male‐specific effects on glutamate accumulation, glymphatic influx, and cognitive performance, we focused subsequent experiments on male mice to elucidate the underlying molecular mechanisms. To determine whether GCPII in hippocampal astrocytes causally contributes to abdominal surgery‐induced cognitive deficits, we engineered GFAP promoter‐driven, miR30‐based shRNA AAV vector to selectively knockdown (KD) endogenous *GCPII* expression in astrocytes (AAV‐GFAP‐EGFP‐mir30‐GCPII), and scrambled control vector (AAV‐GFAP‐EGFP‐mir30‐Scramble) (Figure 4A). AAV‐GFAP‐EGFP‐mir30‐GCPII selectively reduced GCPII expression in vivo, as confirmed by immunostaining in EGFP^+^/GFAP^+^ astrocytes (Figure 4A) and by qPCR (Figure S3A). We also confirmed effective GCPII knockdown in EGFP^+^/Aldh1l1^+^ astrocytes (Figure S3B). AAV‐GFAP‐EGFP‐mir30‐GCPII and AAV‐GFAP‐EGFP‐mir30‐Scramble were injected into the bilateral hippocampus 21 days prior to abdominal surgery, and behavioral testing commenced two days post‐surgery (Figure 4B). Hippocampal astrocyte‐specific GCPII KD ameliorated surgery‐induced deficits in recognition and spatial memory, as assessed by the novel object recognition test, novel location recognition test, and Barnes maze test (Figure 4C). Furthermore, astrocyte‐specific GCPII KD restored BSA‐647–labeled CSF tracer influx into the hippocampus (Figure 4D), demonstrating that astrocytic GCPII mediates surgery‐induced glymphatic impairment. These effects were unlikely to result from nonspecific alterations in body weight and locomotor activity (Figure S3C,D).

**FIGURE 5:**
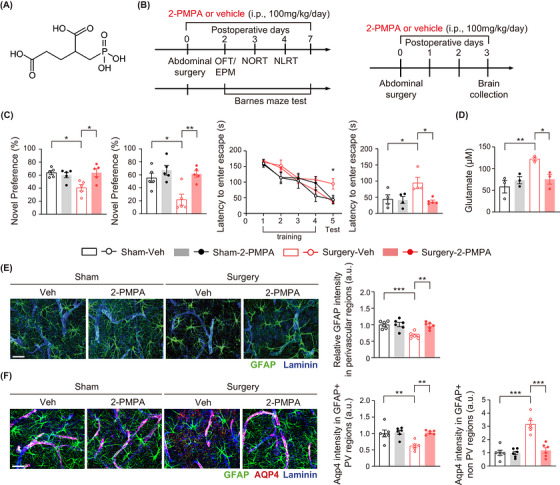
Pharmacological inhibition of glutamate carboxypeptidase II (GCPII) alleviates astrocyte deficits and cognitive impairments in aged male mice following abdominal surgery. (A) Chemical structure of the selective GCPII inhibitor, 2‐PMPA. (B) Schematic diagram of the experimental timeline. Mice received 2‐(phosphonomethyl)‐pentanedioic acid (2‐PMPA) (100 mg/kg, i.p.) or vehicle starting 30 min before abdominal surgery and daily thereafter. Brains were collected on postoperative day 3 for histology. Behavioral analysis was performed using two separate cohorts: one cohort was used for open field test (OFT), elevated plus maze test (EPM), novel object recognition test (NORT), and novel location recognition test (NLRT), and another independent cohort was used for the Barnes maze test from postoperative days 2–7. (C) Left, preference for the novel object in the novel object recognition test, Middle, preference for the novel location recognition test, Right, latency to enter the escape box during the training and test phases of the Barnes maze test for male sham and surgery groups treated with vehicle or 2‐PMPA (sham‐vehicle, *n* = 4; sham–2‐PMPA, *n* = 4; surgery‐vehicle, *n* = 4; surgery–2‐PMPA, *n* = 5). (D) Hippocampal glutamate levels measured 3 days after surgery in vehicle‐ or 2‐PMPA–treated sham and surgery groups (*n* = 3 per group). (E) Left, Immunohistochemistry for glial fibrillary acidic protein (GFAP; green) and Laminin (blue) in hippocampal CA1 region 3 days after surgery; scale bars, 20 µm. Right, quantification of relative GFAP fluorescence intensity in perivascular regions surrounding Laminin^+^ vessels (*n* = 30 cells from five mice per group). (F) Left, representative immunohistochemical images for GFAP (green), aquaporin 4 (AQP4; red), and Laminin (blue) in hippocampal CA1 region; scale bar, 20 µm. Right, quantification of AQP4 signal intensity in GFAP^+^ perivascular (PV) regions and GFAP^+^ non‐PV regions. (*n* = 6 slices in three mice per group). (C–F) *p < 0.05, **p < 0.01, ***p < 0.001, determined two‐way analysis of variance (ANOVA) with Tukey's post hoc test. Data represent mean ± SEM.

We next examined whether pharmacological GCPII inhibition could ameliorate surgery‐induced phenotypes. We have previously reported 2‐(phosphonomethyl)‐pentanedioic acid (2‐PMPA) as a selective GCPII inhibitor (Figure 5A).^70^, ^78^, ^79^ Aged mice received daily 2‐PMPA (100 mg/kg, i.p.) or vehicle, starting on the day of abdominal surgery and continuing throughout the behavioral testing period (Figure 5B). While 2‐PMPA had no impact on body weight or spontaneous locomotor activity in surgery‐exposed mice (Figure S4A,B), 2‐PMPA alleviated surgery‐induced deficits in recognition and spatial memory, as evidenced by improved performance in the novel object recognition test, novel location recognition test, and Barnes maze test (Figure 5C). Furthermore, 2‐PMPA treatment effectively suppressed the abdominal surgery‐induced increase in hippocampal glutamate levels (Figure 5B, D). It also restored GFAP immunoreactivity in perivascular regions and loss of perivascular AQP4 polarization in the hippocampus (Figure 5E, F).

## 4 DISCUSSION

Previous studies have demonstrated that surgery‐induced systemic inflammation triggers microglial activation, cytokine release, and blood‐brain barrier disruption, all contributing to neuroinflammation and cognitive deficits.^3^, ^80^, ^81^, ^82^ Astrocytes are also critical immune cells involved in neuroinflammation.^83^, ^84^ Disruptions in astrocytes‐regulated glutamate homeostasis have also been linked brain diseases, such as multiple sclerosis and brain edema.^31^, ^32^ Nevertheless, their involvement in PND pathophysiology remains underexplored.^85^ Our findings reveal that abdominal surgery induces GCPII activation in hippocampal astrocytes, resulting in male‐specific excess glutamate accumulation and deficits in recognition and spatial memory in aged mice. To our knowledge, this is the first study to identify a sex‐specific astrocyte‐dependent mechanism underlying postoperative cognitive deficits. Interestingly, recent studies have reported that astrocytes in the zona incerta have been implicated in surgery‐induced anxiety,^86^ raising the possibility that region‐specific astrocytic dysfunction may contribute to distinct neurobehavioral consequences of surgery.

Astrocytes maintain extracellular glutamate levels through complex machineries involving metabolic enzymes, glutamate transporters, and ion channels.^87^, ^88^ Importantly, our scRNA‐seq analyses revealed that glutamate signaling pathways are selectively upregulated in a distinct astrocyte subpopulation in males, indicating that astrocytic responses to abdominal surgery are sex dependent. This postoperative astrocyte subpopulation‐specific change may provide a mechanistic link between glutamate dysregulation and male‐specific cognitive deficits. Prior research has indicated hormonal protection in female mice and greater susceptibility of male mice to neuroinflammation and glutamate excitotoxicity, potentially accounting for these differences.^89^, ^90^, ^91^ Of note, sex‐stratified analysis of human *post mortem* hippocampal RNA‐seq data revealed that FOLH1 expression positively correlated with age in both males and females. While significant associations were observed in both sexes, the monotonic age‐associated pattern appeared more consistent in males. This sex difference in age‐associated FOLH1 upregulation may itself contribute to the observed male‐specific vulnerability to postoperative cognitive deficits. Future studies examining inflammatory responses, astrocyte function, and hormonal influences will be important to provide deeper insights into sex differences in PND vulnerability.

The glutamate elevation can alter AQP4 polarization.^42^ AQP4 is essential for glymphatic clearance, and its mislocalization may impair CSF and interstitial fluid exchange. Consistent with this, we demonstrate that abdominal surgery induces male‐specific impairment of glymphatic influx, linking astrocytic glutamate dysregulation to glymphatic dysfunction. A previous study has reported altered AQP4 expression in a mouse model of Alzheimer's disease after tibial fracture surgery,^82^ supporting the notion that perioperative inflammatory responses can disrupt astrocytic function. Our findings extend these observations by identifying sex‐specific vulnerability of the glymphatic system, a feature that may be particularly relevant to postoperative cognitive deficits. It is of interest to further investigate why the glymphatic activity in the hippocampus is uniquely sensitive to abdominal surgery. It should be noted that glymphatic tracer experiments in male and female mice were conducted as independent cohorts using two different tracers, and potential batch effects on absolute tracer signal cannot be excluded. Therefore, direct comparison of baseline glymphatic function between sexes is not feasible in the present dataset. Although a study has reported no sex difference in glymphatic influx in healthy mice,^92^ sex differences in baseline glymphatic function remain insufficiently explored by independent groups, and this represents an important area for future investigation. Additionally, our glymphatic tracing analysis was limited to the 30‐minute peak influx window, and a full kinetic characterization across multiple timepoints would provide deeper mechanistic insight into the temporal dynamics of surgery‐induced glymphatic impairment.

Our study demonstrates increased GCPII enzymatic activity in hippocampal astrocytes following abdominal surgery, leading to excessive hippocampal glutamate accumulation. We found that hippocampal astrocyte‐specific GCPII knockdown can effectively mitigate postoperative cognitive deficits. Pharmacological inhibition of GCPII using the selective inhibitor 2‐PMPA normalized glutamate levels, restored AQP4 polarization in perivascular astrocytes, and ameliorated cognitive deficits. GCPII inhibition has previously shown beneficial cognitive outcomes in rodent models of neuroinflammatory diseases, as well as in aged non‐human primates,^79^, ^93^, ^94^ underscoring its translational potential. It should be noted that systemic administration of 2‐PMPA does not provide cell‐type specificity and, therefore, cannot by itself establish that astrocytic GCPII mediates the observed behavioral effects. Although previous studies including our own have demonstrated that intraperitoneal administration of 2‐PMPA at 100 mg/kg achieves measurable CNS concentrations sufficient to increase brain NAAG levels and inhibit ex vivo GCPII enzymatic activity,^69^, ^70^, ^95^ the contribution of peripheral GCPII inhibition cannot be fully excluded. The 2‐PMPA experiments were therefore intended to provide pharmacological and translational support for GCPII inhibition as a future therapeutic consideration for PND, complementing the mechanistic evidence provided by the astrocyte‐specific GCPII knockdown experiments. Currently, no FDA‐approved pharmacological treatments target PND, and existing management strategies largely focus on mitigating perioperative risk factors.^96^, ^97^, ^98^ Thus, our identification of GCPII as a therapeutic target represents a promising advancement towards addressing a significant clinical need.

In summary, this study elucidates a novel mechanism by which surgery‐induced astrocytic GCPII activation leads to male‐specific glutamate dysregulation, glymphatic dysfunction, and cognitive deficits, highlighting potential therapeutic targets for PND. Further investigation of the molecular and cellular mechanisms underlying these astrocyte‐mediated alterations promises to advance our understanding of PND pathophysiology. Ultimately, this work paves the way for developing novel therapies that can enhance cognitive outcomes and quality of life for older surgical patients.

## CONFLICT OF INTEREST STATEMENT

The authors have declared that no conflict of interest exists. Author disclosures are available in the Supporting Information.

## CONSENT STATEMENT

No human participants were directly recruited for this study. Publicly available, anonymized human transcriptomic data obtained from the Lieber Institute database were used, and no identifiable private information was accessed. Accordingly, additional informed consent was not required.

## Supporting information




Supporting Information

Supporting Information

Supporting Information

Supporting Information

Supporting Information

Supporting Information

Supporting Information

Supporting Information

Supporting Information

Supporting Information
