# Supplementary figures and images for: Glutamate carboxypeptidase II activation in astrocytes mediates glymphatic impairment and cognitive vulnerability in the aging brain following surgery
Source: Alzheimers Dement. 2026 Jul 9;22(7):e71666. doi: 10.1002/alz.71666 (PMC13351328; doi:10.1002/alz.71666)

A

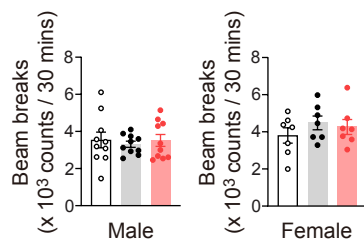

B

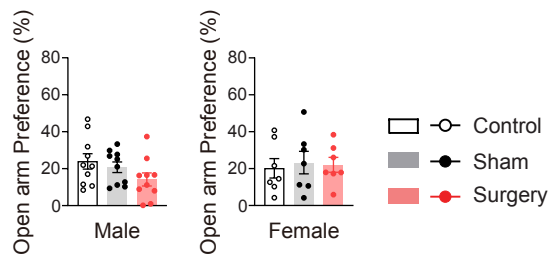

Supplement: Supplementary file 5 — Supporting Information [file ALZ-22-e71666-s006.pdf]

**A**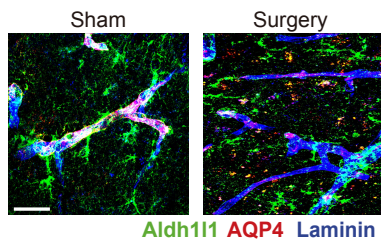**B**

Astrocyte cluster marker gene expression

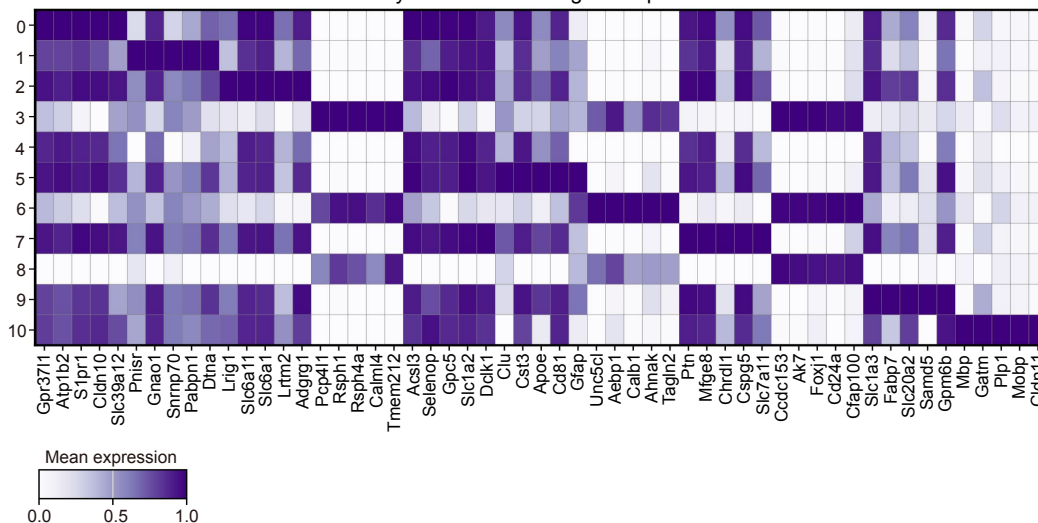**C**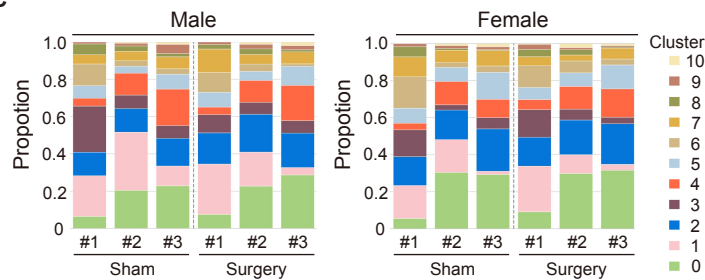

Supplement: Supplementary file 6 — Supporting Information [file ALZ-22-e71666-s003.pdf]

A

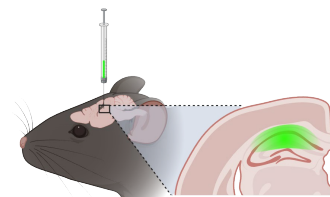

- No injection
- AAV5-GFAP-EGFP-Scrambled control
- AAV5-GFAP-EGFP-GCPII KD
- AAV5-GFAP-EGFP-GCPII KD#2

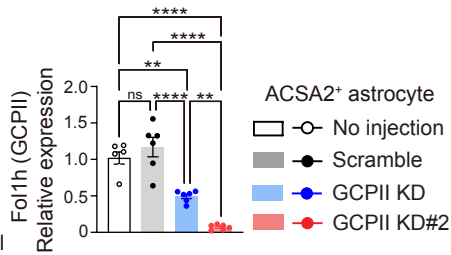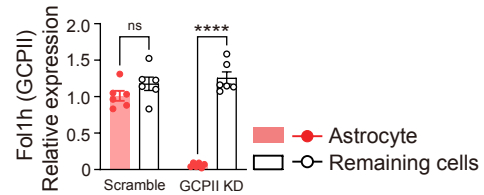

B

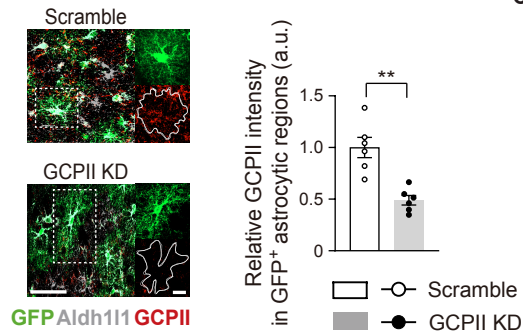

C

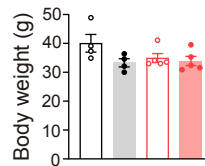

D

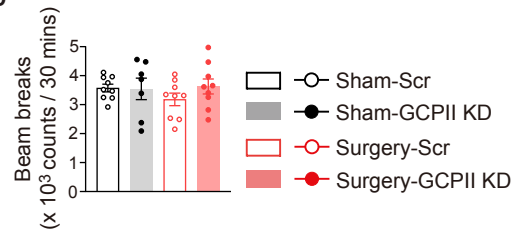

Supplement: Supplementary file 7 — Supporting Information [file ALZ-22-e71666-s010.pdf]

A

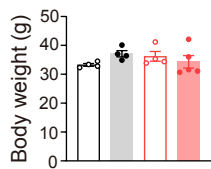

B

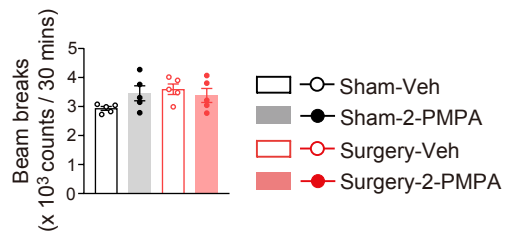

Supplement: Supplementary file 8 — Supporting Information [file ALZ-22-e71666-s004.pdf]
