# Supplementary material for: Glutamate carboxypeptidase II activation in astrocytes mediates glymphatic impairment and cognitive vulnerability in the aging brain following surgery
Source: Alzheimers Dement. 2026 Jul 9;22(7):e71666. doi: 10.1002/alz.71666 (PMC13351328; doi:10.1002/alz.71666)
Supplement: Supplementary file 10 — Supporting Information [file ALZ-22-e71666-s008.pdf]

Supplemental Figure 1. No effect of abdominal surgery on locomotor activity or anxiety-like behavior in aged mice. (**A**) Spontaneous locomotor activity measured by total beam breaks in the open field test. (**B**) Anxiety-like behavior assessed by percentage of time spent in open arms of the elevated plus maze test. (**A, B**) Male (control, n = 10; sham, n = 10; surgery, n = 10) and female (control, n = 7; sham, n = 7; surgery, n = 7) mice. Data represent mean ± SEM.

Supplemental Figure 2. Distribution of astrocyte clusters and their top marker gene expression profiles across samples. (**A**) Representative immunohistochemical images for Aldh1l1 (green), AQP4 (red), and Laminin (blue) in hippocampal CA1 region; scale bar, 20 μm. (**B**) Heatmap of the mean expression of the enriched marker genes for each astrocyte cluster (scaled as log (CPM +1), defined in **Fig. 2D**). (**C**) Proportional distribution of astrocyte clusters (as defined in Figure 2D) across all 12 individual samples in the four conditions (male sham, male surgery, female sham, and female surgery).

Supplemental Figure 3. Efficient Folh1 (GCPII) knockdown in hippocampal astrocytes and no nonspecific effect on body weight, locomotor activity in aged mice. (**A**) Left: Schematic of the unilateral hippocampal injection strategy used to evaluate Folh1 (GCPII) knockdown efficiency, comparing no injection, AAV5-sh-Scramble, AAV5-sh-mFolh1, and AAV5-sh-mFolh1-3 conditions. Middle: Relative *Folh1* (GCPII) mRNA expression in ACSA2$^+$ astrocytes collected from the injected hippocampi of male mice (n = 6 per group). Right: Relative *Folh1* (GCPII) mRNA expression in ACSA2$^+$ astrocytes and remaining non-astrocytic cells collected from the injected hippocampi of male mice (n = 6 per group). ***P < 0.001, **P < 0.01, determined by one-way ANOVA with Tukey's post hoc test. (**B**) Left, representative images of the hippocampal CA1 region from scramble and GCPII KD mice. Images are shown as GFP (green)/Aldh1l1 (gray)/GCPII (red) with high-magnification views

highlighting individual GFP-positive astrocytes; scale bars, 20 μm and 10 μm. Right, quantification of relative GCPII fluorescence intensity in GFP^+ astrocytic regions. (n = 6 slices from 3 mice per group). **P < 0.01, determined by unpaired two-tailed Student's *t*-test. (**C**) Body weight comparison between sham and surgery mice treated with scrambled control or GCPII knockdown (Sham-Scr, n = 4; Sham-GCPII KD, n = 4; Surgery-Scr, n = 5; Surgery-GCPII KD, n = 5). (**D**) Spontaneous locomotor activity (total beam breaks) in open field test assessed in sham and surgery mice treated with scrambled control or GCPII knockdown (Sham-Scr, n = 9; Sham-GCPII KD, n = 7; Surgery-Scr, n = 9; Surgery-GCPII KD, n = 9). Data represent mean ± SEM.

Supplemental Figure 4. No effect of 2-PMPA treatment on body weight and spontaneous locomotor activity in aged mice following abdominal surgery. (**A**) Body weight comparison in sham and surgery mice treated with vehicle (Veh) or 2-PMPA (n = 5 per group). (**B**) Spontaneous locomotor activity (total beam breaks) in open field test in sham and surgery mice treated with Veh or 2-PMPA (n = 5 per group). Data represent mean ± SEM.
